# Synergy through integration of digital cognitive tests and wearable devices for mild cognitive impairment screening

**DOI:** 10.3389/fnhum.2023.1183457

**Published:** 2023-04-18

**Authors:** Aoyu Li, Jingwen Li, Dongxu Zhang, Wei Wu, Juanjuan Zhao, Yan Qiang

**Affiliations:** ^1^College of Information and Computer, Taiyuan University of Technology, Taiyuan, China; ^2^School of Computer Science, Xijing University, Xian, China; ^3^Department of Clinical Laboratory, Affiliated People’s Hospital of Shanxi Medical University, Shanxi Provincial People’s Hospital, Taiyuan, China

**Keywords:** mild cognitive impairment, cognitive assessment, screening tool, wearable devices, touchscreen

## Abstract

**Introduction:**

Advances in mobile computing platforms and the rapid development of wearable devices have made possible the continuous monitoring of patients with mild cognitive impairment (MCI) and their daily activities. Such rich data can reveal more subtle changes in patients’ behavioral and physiological characteristics, providing new ways to detect MCI anytime, anywhere. Therefore, we aimed to investigate the feasibility and validity of digital cognitive tests and physiological sensors applied to MCI assessment.

**Methods:**

We collected photoplethysmography (PPG), electrodermal activity (EDA) and electroencephalogram (EEG) signals from 120 participants (61 MCI patients, 59 healthy controls) during rest and cognitive testing. The features extracted from these physiological signals involved the time domain, frequency domain, time-frequency domain and statistics. Time and score features during the cognitive test are automatically recorded by the system. In addition, selected features of all modalities were classified by tenfold cross-validation using five different classifiers.

**Results:**

The experimental results showed that the weighted soft voting strategy combining five classifiers achieved the highest classification accuracy (88.9%), precision (89.9%), recall (88.2%), and F1 score (89.0%). Compared to healthy controls, the MCI group typically took longer to recall, draw, and drag. Moreover, during cognitive testing, MCI patients showed lower heart rate variability, higher electrodermal activity values, and stronger brain activity in the alpha and beta bands.

**Discussion:**

It was found that patients’ classification performance improved when combining features from multiple modalities compared to using only tablet parameters or physiological features, indicating that our scheme could reveal MCI-related discriminative information. Furthermore, the best classification results on the digital span test across all tasks suggest that MCI patients may have deficits in attention and short-term memory that came to the fore earlier. Finally, integrating tablet cognitive tests and wearable sensors would provide a new direction for creating an easy-to-use and at-home self-check MCI screening tool.

## 1. Introduction

Health conditions such as memory loss, frequent repetitive questioning, and geographic orientation impairment affect the daily lives of many older adults (Knopman and Petersen, 2014). These cognitive issues usually are attributed to “normal for age” (Breitner, 2014). However, when these initial signs occur more frequently, they may indicate something more substantial, such as mild cognitive impairment (MCI). MCI is an intermediate state between normal aging and dementia in which one or more of the corresponding cognitive impairments in language, memory, attention, visuospatial and executive functions are present (Burns and Zaudig, 2002). A recent study showed that ~22.7% of the total United States population might suffer from some form of MCI (Rajan et al., 2021). In addition, given that many of the early symptoms in MCI are insidious, patients may confuse cognitive impairment with normal aging, leading to a delay in the actual diagnosis and exacerbating the progression to dementia.

Early detection of dementia has been shown to allow interventions to slow the evolution of the disease, such as physical activity (Groot et al., 2016) and pharmacological interventions (Hansen et al., 2008). However, in the clinical setting, commonly available cognitive screening tests [e.g., the Mini-Mental State Examination (MMSE) test (Folstein et al., 1975); the Montreal Cognitive Assessment (MoCA) test (Nasreddine et al., 2005)] and other screening tools (e.g., cerebrospinal fluid examination and magnetic resonance imaging) are time-consuming, invasive or not readily available. Furthermore, these assessments are subjective and require constant attention from managers. Thus, the effective diagnosis of MCI remains one of the most difficult challenges in geriatric psychiatry (Gosztolya et al., 2019).

Evidence is mounting that changes in cognition, behavior, sensation and movement in patients with Alzheimer’s disease (AD) may manifest years earlier than clinical symptoms (Sperling et al., 2014). Diagnosing MCI and dementia based on clinical features alone is challenging and relatively unreliable (Grässler et al., 2021). In recent years, with the rapid development of computer technology, computer-aided diagnostic techniques have contributed tremendously positively to improving diagnostic accuracy, reducing missed diagnoses, and increasing efficiency (Chen et al., 2022a; Yu et al., 2022). In the early screening of neurodegenerative diseases (e.g., dementia), several researchers have attempted to use consumer-grade mobile and wearable technologies to explore effective digital biomarkers. For example, Müller et al. (2019) captured a large number of kinematic features of participants during a digital clock drawing test. They found that dwell time in the air appeared to be a distinctive feature between MCI patients and healthy individuals. Later, Ehsani et al. (2020) assessed the uncertainty of elbow angle and angular velocity in older adults while wearing a tri-axial gyroscope performing an upper limb dual task, highlighting the potential of entropy of elbow angular velocity in detecting cognitive impairment. Furthermore, Ladas et al. (2014) discovered that blink rates per minute were higher in MCI patients than in healthy controls (HC), suggesting that eye blink rates would be promising as one of the potential biomarkers of MCI. Finally, Jonell et al. (2021) used nine sensors to capture data on participants’ behavioral and physiological signals during clinical interviews and identified head temperature changes and mapping gap length as novel digital biomarkers perhaps associated with early AD diagnosis. Thus, mobile applications and continuous passive sensor data may improve individuals’ early detection and monitoring, and provide more effective clinical decision-making.

With significant technological advances in the ubiquitous availability of convenient devices and wearable sensors, continuous monitoring of patients and their daily activities has become possible. Combining low-cost and non-invasive methods of measuring an individual’s physical signs with a game-based screening test for serious cognition will allow older adults to detect and track cognitive decline with minimal disruption and burden. This paper aims to explore the validity and feasibility of extracting features from physiological signals (e.g., PPG, EDA, and EEG) and digital cognitive parameters to assess MCI. Specifically, subjects’ physiological data are recorded at rest and while performing a cognitive task. The digital cognitive parameters consist of time and score, while features of the physiological data are extracted from several modalities, time domain, frequency domain, time-frequency domain and statistical. After obtaining the optimal feature subset by a feature selection algorithm, we compared the classification performance of the feature subset in single-mode and fused multi-mode. We hypothesized that features extracted from all physiological modalities fused with cognitive parameters would be the best for classification. Finally, five machine learning classification algorithms, including k-Nearest Neighbor (kNN), Decision Tree (DT), Random Forest (RF), Naive Bayes (NB) and XGBoost (GBDT), were used to classify healthy individuals and MCI patients. In particular, their classification accuracy was used as a decision weight for model predictions, considering the differences in screening performance for each cognitive test.

## 2. Materials and methods

### 2.1. Participants

A total of 120 participants (62 females and 58 males) were recruited consecutively. Participants met the following criteria: (1) normal or corrected normal hearing and vision; (2) age > 65 years; (3) completed the MMSE test; (4) completed the MoCA test; and (5) were able to do moderate exercise and had no physical disability. The MCI group comprised 61 subjects who scored below 26 on the MoCA scale. Clinical interviews confirmed that healthy individuals had no neurological or psychiatric history and showed no signs of cognitive decline. In addition, all subjects had not previously performed these tasks and completed all test items. Table 1 summarizes the clinical statistical information for the 120 participants.

**Table 1:** Clinical and demographic characteristics.

	HC (*n* = 59)	MCI (*n* = 61)	Value of *p*
Age avg. (std)	67.90 (6.185)	70.98 (5.846)	**0.025**
Gender (F[%]/M[%])	28[47]/31[53]	34[56]/27[44]	0.273
Education level avg. (std)	7.87 (2.839)	5.05 (3.514)	**<0.001**
Sleep quality avg. (std)	6.44 (0.821)	6.12 (1.122)	0.159
Exercise habits (Y[%]/N[%])	48[81]/11[19]	35[57]/26[43]	**0.012**
MoCA avg. (std)	27.10 (1.119)	23.20 (1.436)	**<0.001**
MMSE avg. (std)	28.77 (0.872)	25.05 (1.482)	**<0.001**

The bold values mean that there is a significant difference between the two groups.

### 2.2. Experimental equipment and procedure

In this study, we used an iPad 2019, Empatica E4 and MUSE 2 to collect digital cognitive parameters and physiological signals from participants at rest and in the task state. All cognitive tests were presented on the iPad 2019 (7th generation, 3GB/128GB, 10.2″, 2,160 × 1,620 pixel touchscreen), and Apple Pencil to perform drawing-related tasks. The tablet records the start and end times of each test in real time, providing the basis for subsequent synchronization of physiological data. The Empatica E4 is a watch-like multi-sensor device that measures EDA, PPG, skin temperature and accelerometer data. It is small, lightweight and comfortable to wear, making it particularly suitable for unobtrusive, continuous monitoring in the cognitive screening of older adults. We charge and synchronize the E4 to a laptop computer before use. The data is typically stored on the E4 and then transferred to the computer for processing. The MUSE 2 is a wire-free, portable, wearable, and flexible EEG headband widely used in meditation and research (Cannard et al., 2021; Hunkin et al., 2021; Chai et al., 2023). It contains five electrodes, two of which are frontal electrodes (AF7 and AF8), two others are temporal electrodes (TP9 and TP10) and a reference electrode located at the Fpz position. The MUSE headband uses Bluetooth technology to send data through the Muse monitor at a sampling rate of 256 Hz.

**Figure 1:**
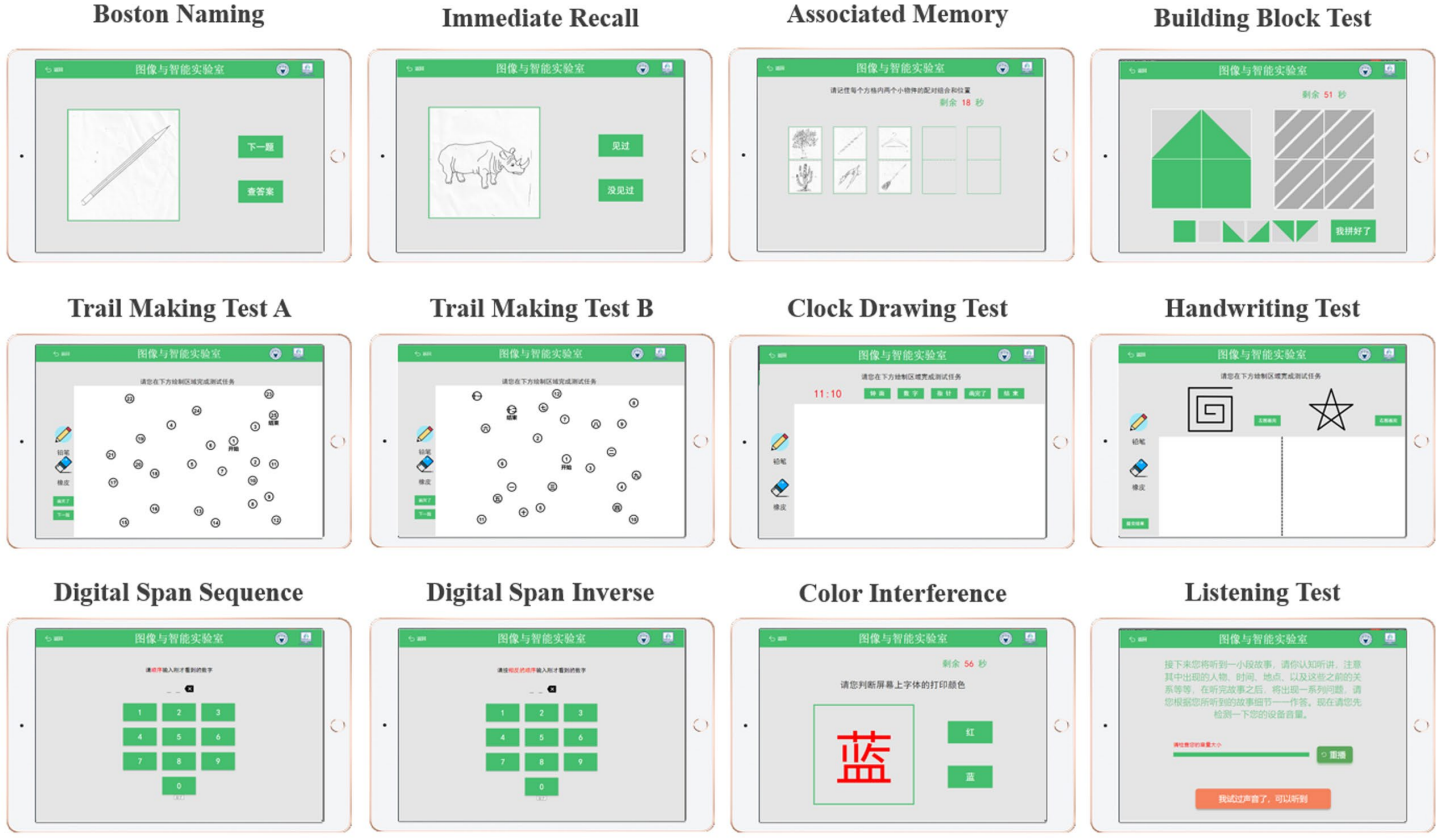
Tablet-based digital cognitive testing.

**Figure 2:**
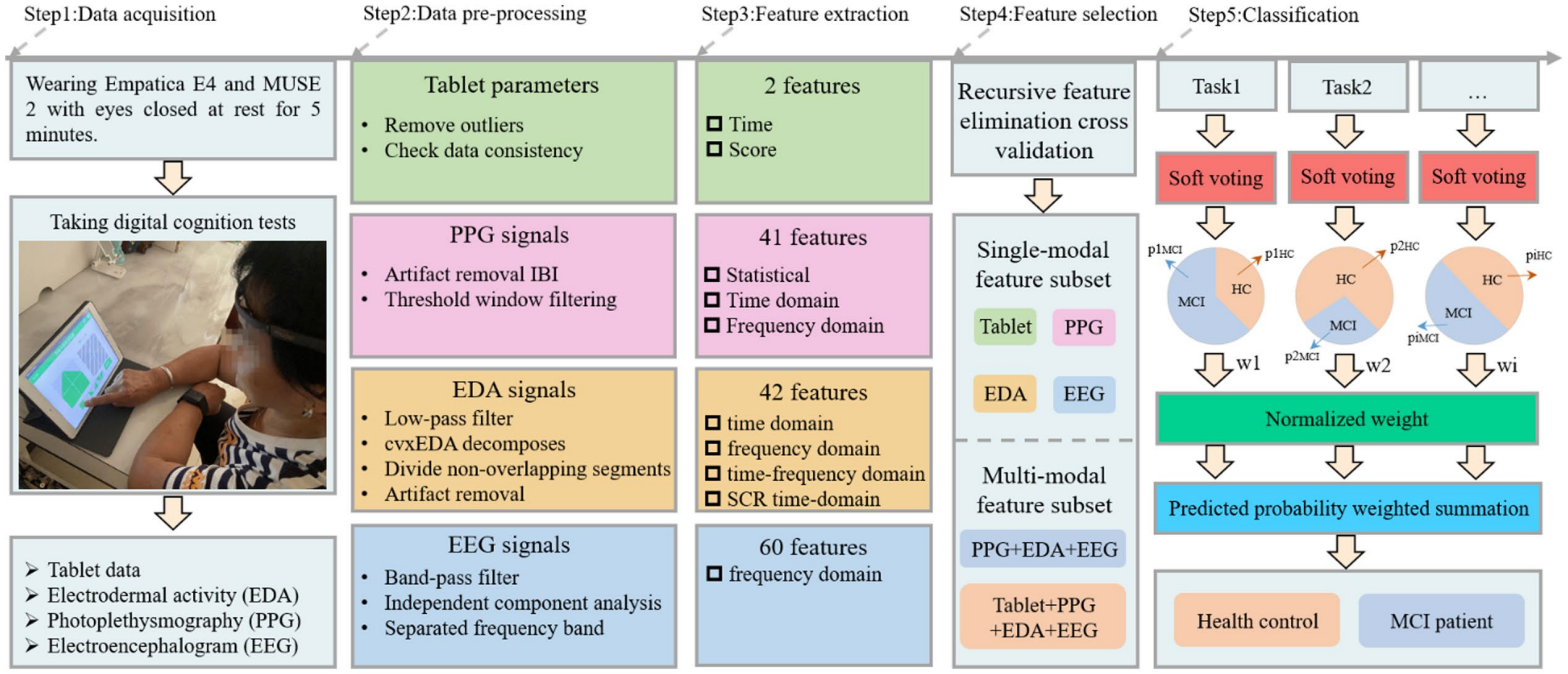
The proposed MCI detection framework for multi-source data.

The cognitive test was developed through discussions between two neurologists, two nurses and three engineers from our team. Specifically, it includes Boston naming, immediate recall, associated memory, building block test, trail making test A, trail making test B, clock drawing test, handwriting test, digital span sequence, digital span inverse, color interference and listening test, as shown in Figure 1. The battery assessed various cognitive abilities, including verbal fluency, memory, attention, listening, visuospatial and executive function. Before the experiment begins, the experimenter will explain the procedure to the participants and obtain their written consent. Once consent was obtained, the experimenter would place the MUSE on the subject’s forehead and the Empatica on the wrist of the subject’s non-dominant hand (Boucsein, 2012; Gashi et al., 2019). The subjects were asked to sit comfortably, fully relaxed, and record physiological signals with their eyes closed for 5 min. Next, subjects began performing cognitive tasks with real-time access to wearable device physiological signals and tablet cognitive data, as shown in Step 1 (Figure 2). In particular, for cognitive tasks involving drawing, the experimenter would provide some assistance (e.g., fixing the tablet) during the drawing process to minimize the participant’s non-dominant hand involvement.

### 2.3. Data processing and analysis

We draw on the computer-aided diagnostic medical image analysis process, i.e., from dataset to preprocessing, feature extraction, feature selection, and classification (Li et al., 2022; Hu et al., 2023) for the acquired cognitive and physiological data. The details are described below.

#### 2.3.1. Data pre-processing

HRV describes the irregularity between two consecutive heartbeats by measuring variations known as the RR interval or interbeat interval (IBI). It is pleasing to note that IBI data can be easily obtained from the participant’s E4 wristband. By pre-processing the IBI, the reliability of HRV can be effectively improved. First, we removed noticeable artifacts from the IBI series according to the rules for detecting artifacts in existing studies (Acar et al., 2000; Karlsson et al., 2012). Second, for those missing heartbeat data not identified by the measurement device, we increase the reliability of the HRV measurement by defining a threshold standard to remove imperfect windows, as suggested by Föll et al. (2021). Thus, if a time window t satisfies the following inequality, it will not be discarded.


(1) $$thresh\cdot \frac{L}{\mu_{IBIt}}<N_{t}$$

where *N_t_* denotes the number of valid heart beats detected in window *t*, *L* denotes the epoch width (window length) in seconds, *μ_IBIt_* denotes the average IBI (in seconds) in window t and thresh ∈ [0, 1]. In summary, a higher threshold setting will result in a higher proportion of detected heartbeats relative to the desired amount of expected heartbeats. Here, we set the epoch width to 180 and the threshold to 0.2.

Autonomous activation of human sweat glands causes skin conductance changes and is a phenomenon commonly referred to as EDA. To improve the obtained EDA signal quality, we followed the same preprocessing steps suggested by Boucsein (2012), Gashi et al. (2019), Hassib et al. (2017), and Martínez-Rodrigo et al. (2017): (1) A first-order Butterworth low-pass filter with a cut-off frequency of 0.6 Hz is used to remove high-frequency noise fluctuations from the signal; (2) EDA signal is further decomposed into tonic and phase components using the cvxEDA method proposed in the literature (Greco et al., 2015); (3) Given that the SCR response duration is between 1 and 5 s, we divide the EDA series into 5 s non-overlapping segments and extract most of the features suggested by existing literature (Gashi et al., 2019; Shukla et al., 2019) to describe shape artifacts; (4) Machine learning was used to identify shape artifacts, thereby effectively distinguishing between standard EDA signals and artifacts.

The EEG signal is an overall reflection in the cerebral cortex or scalp surface from the electrophysiological activity of brain nerve cells. As a typical signal in body sign signals, it contains much neurophysiological information. Owing to its susceptibility to the state of contact between the scalp and the sensor and to interference from environmental noise, we followed several routine pre-processing steps as recommended by the OHBM COBIDAS MEEG committee (Pernet et al., 2020). First, the recorded EEG data were band-pass filtered to 1–45 Hz using the egfiltfft function in the EEGLab toolbox. Then, independent component analysis (ICA) was applied to each channel’s signal to detect and remove eye movements, muscle artifacts, channel noise and outlier data segments. Subsequently, the remaining data epochs were manually checked to remove data segments with significant artifacts or drowsiness features that were not automatically removed. Finally, the EEG signal was separated into five typical bands, namely the delta band (1–4 Hz), theta band (4–8 Hz), alpha band (8–13 Hz), beta band (13–30 Hz) and gamma band (13–45 Hz), employing a second-order Butterworth band-pass filter.

The tablet-based digital cognitive test records hand movements and cognitive performance in older adults. Features extracted from these data will be used as digital biomarkers that may distinguish healthy individuals from those with mild cognitive impairment. Therefore, the raw data needs to be cleaned before features can be extracted, including removing outliers and checking data consistency.

#### 2.3.2. Feature extraction and selection

After pre-processing, the next step was to extract features from the wearable sensor signals and tablet data to classify healthy individuals and MCI patients, as shown in Step 3 (Figure 2). Three frequency domain features, namely mean power (MP), spectral entropy (SE) and asymmetry index (AI) were extracted from the EEG signal and these features have been applied to MCI detection (Luckhaus et al., 2008; Bruña et al., 2012; Martin et al., 2022). MP is derived by calculating the mean of the absolute power in each band. SE is a measure of unpredictability and disorder associated with the spectrum of a signal, and a higher SE indicates a higher level of complexity. The MP and SE have 20 features each, i.e., four channels in the MUSE headband, each with five bands. AI includes Differential Asymmetry (DASM), which is the absolute power difference between each band of the left and right hemisphere asymmetric channels (TP9 & TP10 and AF7 & AF8), and Reasonable Asymmetry (RASM), which is the absolute power ratio between each band of the left and right hemisphere asymmetric channels (TP9 & TP10 and AF7 & AF8). Ten feature values were obtained from the EEG data for each DASM and RASM feature set. The final total number of extracted EEG features was 60. For the EDA and PPG signals collected from the E4 wristband, we used FLIRT (Föll et al., 2021) to extract 41 HR or HRV features (these features belong to the statistical, time domain, and frequency domain) and 42 EDA features (these features belong to the time domain, frequency domain, time-frequency domain and SCR time-domain). Finally, the time spent and scores obtained by the participants for each test were extracted from the tablet. The specific features can be found in the Supplementary material.

Regarding feature selection, we use recursive feature elimination cross-validation (RFECV) to separate the best subset of features from each physiological signal, enabling the classification model to maintain accuracy while reducing the computational cost. The algorithm is divided into two phases: (1) creating the model through iterations, eliminating the worst features or retaining the best features in each iteration. Subsequent iterations use the unselected features from the previous modeling to build the next model until all features are used. The features are ranked according to the order in which they are retained or rejected. (2) Different numbers of features are sequentially selected from the ranked feature set for cross-validation, and the number of features with the highest mean score is determined by comparison to obtain the best feature subset. In addition, the RFECV we use for single-mode and multi-mode data is performed independently with a 10-fold cross-validation.

#### 2.3.3. Classification

In this study, different cognitive tests may affect the MCI diagnosis and correspond to different classification accuracies, so we proposed a weighted soft voting strategy for classification, as shown in Step 5 (Figure 2). The fused base classifiers in the soft voting principle include kNN, DT, RF, NB and GBDT. Here, the tree count of RF was set to 100, NB is Gaussian Bayes, and the tree count, learning rate, and booster of XGBoost are 100, 0.1, and gbtree, respectively. Cross-validation is 10-fold cross-validation, where all samples are divided into 10 equal parts and any first part is treated as test data. We normalized each test cross-validation score and used them as predictive model weights (e.g., w,w1,2,…,wi). In other words, identifying a subject as MCI can be derived from the following formula.


(2) $$P_{MCI}=\overset{12}{\underset{i=1}{\sum }}pi_{MCI}\times wi$$

(3) $$P_{HC}=\overset{12}{\underset{i=1}{\sum }}pi_{HC}\times wi$$

$P_{MCI}$ denotes the probability that the predicted individual belongs to MCI, $pi_{MCI}$ is the probability that the *i*-th test soft-vote predicts an individual to be MCI, and wi is the normalized value of the *i*-th test soft-vote cross-validation score. The same is true for $P_{HC}$, $pi_{HC}$. Individual categories are predicted by comparing the magnitude of $P_{MCI}$ and $P_{HC}$. Finally, in line with most evaluation metrics in the literature (Zhang J. et al., 2021; Chen et al., 2022b), we use accuracy, precision, recall, and F1 score to measure classification performance.

## 3. Results

For the statistical analysis of demographic characteristics, digital cognitive parameters and physiological sensor data, the Kolmogorov–Smirnov test was used to test for normality. Welch correction was applied to the uneven variance data. A t-test was used to compare differences between MCI patients and healthy individuals. The non-parametric Mann–Whitney test was applied to assess group differences in the score variables and the Hodges-Lehmann estimator estimated confidence intervals, as the variable did not follow a normal distribution. In addition, for age, education, MoCA, MMSE and sleep quality, statistical descriptions were performed using means (standard deviations). However, gender and exercise habits were categorical variables described by percentages, and Pearson’s chi-square test was used to detect differences between groups. For all tests, the level of statistical significance was set at *p* < 0.05.

### 3.1. Digital cognitive tests performance in healthy individuals and MCI patients

**Figure 3:**
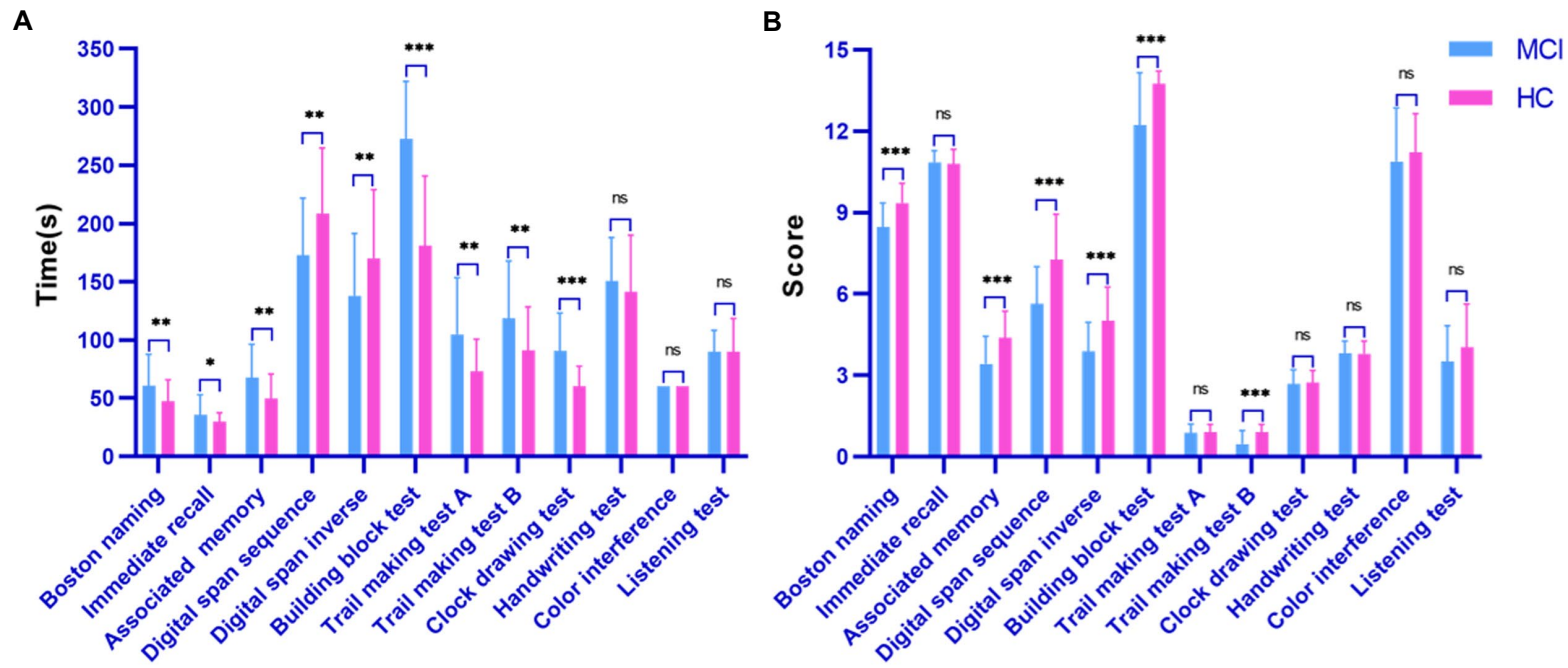
Performance of the surveyed group in the digital cognitive test. **(A)** Time cognitive parameter; **(B)** Score cognitive parameter.

Figure 3 shows the statistical comparison results of the time and score features among the investigated groups (MCI patients and healthy controls) during the digital cognitive test. We found that both features performed well in distinguishing MCI patients from healthy individuals. Specifically, except for three tests (handwriting test, color interference and listening test), there was a significant difference in finishing time between healthy individuals and MCI patients (all *p* < 0.05; Figure 3A). The MCI group usually took longer to recall, draw and drag than the control group. Interestingly, in the digital span test, healthy individuals could correctly recall previous digits and move on to the next level, increasing their time overhead. However, in terms of score features, the two groups differed significantly on Boston naming, associated memory, digital span sequence, digital span inverse, building block test, and trail making test B (all *p* < 0.001; Figure 3B). Finally, the color interference and listening test were less effective in screening MCI patients and healthy individuals, which may indicate that MCI patients are aligned with healthy individuals in their ability to inhibit habitual behavior and auditory memory.

### 3.2. Physiological signals performance in healthy individuals and MCI patients

**Figure 4:**
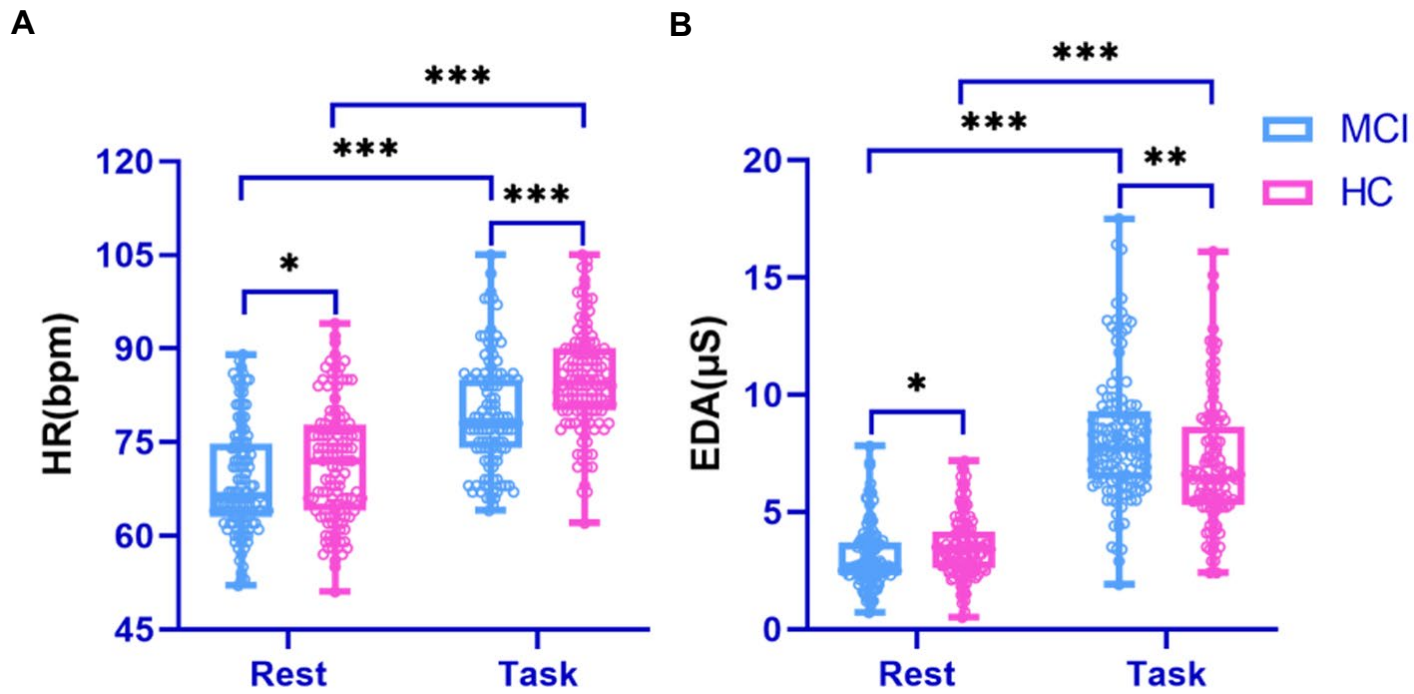
Performance of physiological signals in the surveyed group during rest and cognitive testing. **(A)** HR signal; **(B)** EDA signal.

**Figure 5:**
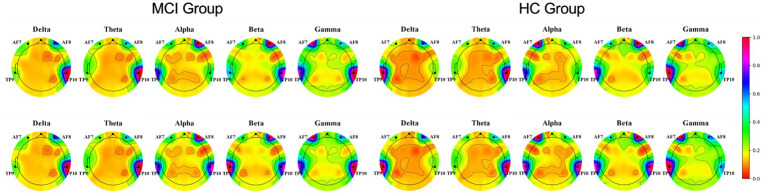
Brain activity visualization in different EEG bands at power spectral density during two phases of the experiment (Row 1: rest-state, Row 2: during cognitive testing).

Physiological signals obtained by Empatica E4 at rest and while performing a cognitive task were analyzed using paired *t*-tests. We found significant differences between the HR and EDA data obtained by the investigated groups during the experiment’s two phases (all *p* < 0.001; Figure 4). It is also evident from the box plots that MCI patients exhibited lower HRV and higher EDA values during the test, which may reflect dysautonomia and impaired health in MCI patients (Rossini et al., 2008). Furthermore, Figure 5 shows a brain activity visualization of participants at rest and in the task state from the power spectral density. A brain map in red describes intense brain activity, while orange indicates weak cortical activation. We can conclude from the experimental results: (1) the MCI group had increased power spectral density at slower frequencies (i.e., delta and theta) compared to the control group (i.e., the MCI group showed yellow brain maps while the HC group showed orange brain maps in the delta and theta bands) and (2) the electrodes in the alpha and beta bands during the task had visually different brain activity compared to the resting state, which was evident in both the healthy control and MCI groups (i.e., MCI: alpha-TP10, beta-TP9; HC: alpha-TP10, beta-TP10).

### 3.3. Predictive value of the digital cognitive tasks

We examined the classification performance of each task in the digital cognitive test, as shown in Table 2. The results show that digital span inverse had the highest detection accuracy of 84.4%. As far as precision is concerned, digital span inverse also had the best results. That is, 85.7% of all predicted MCI patients in digital span inverse were actual MCI patients. Digital span sequence achieved the best recall, indicating that this task could correctly detect 86.4% of real MCI patients. The F1 score takes into account both accuracy and recall. Digital span inverse achieved the best result of 85.2% in this metric, demonstrating the better overall performance of digital span inverse. Furthermore, to explore which physiological features were more prominent in distinguishing the MCI and HC groups, we selected features ranked 3, 3, and 2 (HRV, EDA, and EEG) in importance from the subset of best features for each cognitive test. The experimental results involved three aspects: (1) SDNN, pNN50 and LF occur more frequently in HRV. In other words, lower SDNN indices and PNN50 indicate that MCI patients may be vulnerable to autonomic and parasympathetic dysfunction (Xue et al., 2022). However, LF power performed more prominently on memory and attention-related tests (Nicolini et al., 2020), further strengthening the classification performance of the Digital Span (which measures a person’s attention and short-term memory capacity); (2) Phasic signals in EDA features, particularly those derived from SCR components (transient, faster fluctuations in skin conductance) are most prominent, such as phasic kurtosis, amp (mean amplitude of the SCR peaks) and decay time (mean of the SCR peaks decay time). Compared to healthy individuals, MCI patients typically show higher amp and longer decay time, indicating that they are more reactive to external stimuli and less able to recover from stress; (3) Interestingly, the EEG features of each test are mainly focused on the alpha and beta bands. The alpha and beta bands contribute to the perfect oscillation of human consciousness in cognitive reasoning, computation, reading, communication and thought states, suggesting that touchscreen-based tests can have a better stimulating and arousing effect on older adults.

**Table 2:** Performance comparison of classification of selected feature subsets for 12 digital cognitive tests.

Tasks	Acc	Pre	Rec	F1	Tablet	HRV	EDA	EEG
T1	0.756	0.765	0.841	0.801	T S	H^b^ H^d^ H^h^	E^a^ E^c^ E^d^	G^c^ G^e^
T2	0.683	0.710	0.805	0.755	T	H^a^ H^c^ H^j^	E^c^ E^e^ E^j^	G^c^ G^f^
T3	0.756	0.768	0.836	0.801	T S	H^a^ H^b^ H^j^	E^d^ E^k^ E^h^	G^e^ G^g^
T4	0.843	0.843	0.828	0.836	T S	H^a^ H^g^ H^i^	E^f^ E^i^ E^j^	G^h^ G^o^
T5	0.729	0.738	0.745	0.742	T	H^b^ H^d^ H^h^	E^c^ E^e^ E^h^	G^c^ G^d^
T6	0.786	0.780	0.857	0.816	T S	H^a^ H^d^ H^e^	E^c^ E^i^ E^k^	G^g^ G^k^
T7	0.783	0.789	0.862	0.824	T	H^a^ H^d^ H^j^	E^f^ E^j^ E^k^	G^g^ G^m^
T8	0.734	0.750	0.825	0.786	/	H^a^ H^d^ H^l^	E^a^ E^c^ E^j^	G^a^ G^d^
T9	0.816	0.822	**0.864**	0.842	T S	H^g^ H^j^ H^l^	E^d^ E^e^ E^h^	G^i^ G^l^
T10	**0.844**	**0.857**	0.847	**0.852**	T S	H^a^ H^d^ H^j^	E^g^ E^i^ E^k^	G^j^ G^n^
T11	0.654	0.687	0.782	0.731	/	H^b^ H^e^ H^g^	E^d^ E^f^ E^j^	G^b^ G^c^
T12	0.742	0.764	0.773	0.769	/	H^a^ H^h^ H^k^	E^h^ E^i^ E^k^	G^j^ G^p^

The bold values mean the largest attribute’s values in the table. T1: Boston naming; T2: immediate recall; T3: associated memory; T4: building block test; T5: trail making test A; T6: trail making test B; T7: clock drawing test; T8: handwriting test; T9: digital span sequence; T10: digital span inverse; T11: color interference; T12: listening test; H^a^: SDNN; H^b^: RMSSD; H^c^: NN50; H^d^: pNN50; H^e^: sum of the IBIs; H^f^: skewness; H^g^: peaks; H^h^: entropy; H^i^: VLF; H^j^: LF H^k^: HF; H^l^: LF/HF ratio; E^a^: tonic rms; E^b^: tonic kurtosis; E^c^: phasic kurtosis; E^d^: tonic perm_entropy; E^e^: phasic perm_entropy; E^f^: tonic skewness; E^g^: phasic skewness; E^h^: rise time; E^i^: max derive; E^j^: amp; E^k^: decay time; G^a^: MP_theta_AF7; G^b^: MP_theta_AF8; G^c^: MP_alpha_AF7; G^d^: MP_alpha_AF8; G^e^: MP_alpha_TP9; G^f^: MP_alpha_TP10; G^g^: MP_beta_AF7; G^h^: MP_beta_AF8; G^i^: MP_beta_TP9; G^j^: MP_beta_TP10; G^k^: SE_alpha_AF7; G^l^: SE_alpha_TP9; G^m^: SE_beta_TP9; G^n^: DASM_alpha_AF8; G^o^: DASM_beta_AF7; G^p^: RASM_beta_AF7; T: time; S: score.

### 3.4. Predictive value of the classification framework

We compared the classification performance of the proposed classification framework under different classifiers for cognitive features from tablets, physiological features from wearable devices, and their combined features, as shown in Tables 3, 4. As expected, the combined features of all modalities yielded the highest classification accuracy. In particular, the highest classification accuracy (88.9%), precision (89.9%), recall (88.2%) and F1 values (89.0%) were obtained using the weighted soft voting strategy (Table 4). Furthermore, we compared it with digital cognitive tests and wearable sensors used for MCI detection in recent years, as shown in Table 5. The results show that our cognitive system outperformed recent studies in correctly identifying MCI patients, achieving an accuracy rate of 85.1%. Features fusing HRV, EDA and EEG signals were also the best when used to differentiate healthy individuals from MCI patients, achieving an accuracy of 86.2%, indicating that the use of fused features from multimodal data is more effective than unimodal data for classifying MCI. Similarly, our proposed MCI classification scheme outperformed all these methods by using fused features selected from Tablet, HRV, EDA and EEG signals, achieving a classification accuracy of 88.9%. Finally, the number of subjects participating in the experiment was 120, which is also the highest compared to studies using non-invasive wearable sensors to screen elderly with cognitive impairment.

**Table 3:** Performance comparison of the classification framework with different classifiers for single-modal features from tablets and wearable devices.

Classifier	HRV				EDA				EEG				Tablet			
	Acc	Pre	Rec	F1	Acc	Pre	Rec	F1	Acc	Pre	Rec	F1	Acc	Pre	Rec	F1
kNN	0.724	0.721	0.731	0.726	0.667	0.648	0.728	0.686	0.682	0.685	0.680	0.683	0.753	0.765	0.752	0.759
DT	0.758	0.749	0.771	0.760	0.724	0.755	0.698	0.726	0.735	0.738	0.734	0.736	0.782	0.794	0.809	0.801
NB	0.775	0.752	0.769	0.760	0.733	0.744	0.732	0.738	0.738	0.735	0.740	0.737	0.798	0.801	0.812	0.806
RF	0.860	0.757	0.764	0.760	0.731	0.727	0.744	0.735	0.741	0.740	0.742	0.741	0.801	0.806	0.829	0.817
GBDT	0.794	0.779	0.818	0.798	0.774	0.761	0.793	0.777	0.790	0.789	0.791	0.790	0.836	0.839	0.848	0.843
Soft voting	0.825	0.815	0.828	0.822	0.801	0.800	0.807	0.803	0.823	0.835	0.822	0.829	**0.851**	**0.858**	**0.870**	**0.864**

The bold values mean the largest attribute’s values in the table.

**Table 4:** Performance comparison of a classification framework with different classifiers for multi-modal fusion features of tablets and wearable devices.

Classifier	HRV + EDA + EEG				Tablet+HRV + EDA + EEG			
	Acc	Pre	Rec	F1	Acc	Pre	Rec	F1
kNN	0.775	0.786	0.779	0.783	0.804	0.809	0.801	0.805
DT	0.794	0.807	0.798	0.802	0.825	0.834	0.829	0.832
NB	0.807	0.811	0.820	0.815	0.827	0.845	0.831	0.838
RF	0.816	0.826	0.818	0.822	0.834	0.866	0.841	0.853
GBDT	0.844	0.854	0.848	0.851	0.864	0.870	0.860	0.865
Soft voting	0.862	0.873	0.863	0.868	**0.889**	**0.899**	**0.882**	**0.890**

The bold values mean the largest attribute’s values in the table.

**Table 5:** Diagnostic value of our classification framework compared to existing studies of healthy individuals and MCI patients.

Study, Year	HC/MCI	Equipment	Examination	Modalities	Acc
Müller et al. (2019)	138/137	Microsoft Surface Pro	Clock drawing test	Tablet	0.815
Zhang Y. et al. (2021)	20/41	Huawei M5 tablet	cogSYS	Tablet	0.824
Ntracha et al. (2020)	12/11	Smart Phone	Typing tasks	Phone	0.800
Alharbi et al. (2022)	21/21	CorSense	Resting state	HRV	0.765
Gwak et al. (2018)	35/34	Nonin Onyx 2	Cognitive tests	PPG	0.820
Lee et al. (2022)	22/21	Pico Neo 2, Looxid Link	Cognitive tests	EEG	0.800
This paper	59/61	iPad 2019, Empatica E4, MUSE 2	Cognitive tests	Tablet	**0.851**
				HRV	**0.825**
				EEG	**0.823**
				HRV, EDA, EEG	**0.862**
				Tablet, HRV, EDA, EEG	**0.889**

The bold values mean the largest attribute’s values in the table.

## 4. Discussion

This paper describes a non-invasive and convenient classification approach to facilitate early sign detection in MCI by simultaneously collecting cognitive behavioral and physiological data. To our knowledge, this is a rare study using a mobile app and multimodal wearable sensors (e.g., PPG, EDA and EEG) to assess and monitor physiological variations in MCI patients. Specifically, we fused features extracted from all physiological modalities and digital cognitive parameters, and applied machine learning to classify healthy individuals and MCI patients. The experimental results show that our classification framework works well, achieving 88.9% accuracy, reflecting the synergistic effect of combining digital cognitive tests and physiological sensor recordings in the context of MCI screening. In addition, the cognitive screening process can be carried out portable and without the involvement of a medical specialist, regardless of the test location, thus providing a low-cost and flexible family screening paradigm for the early detection of MCI.

Secondly, regarding cognitive domains, our digital cognitive tests covered multiple dimensions of language, memory, attention, visuospatial and executive function, which are core dimensions included in many existing computerized cognitive assessment tools (Gates and Kochan, 2015; Demeyere et al., 2021). A comparison of categorical performance on 12 cognitive tests found that the surveyed group had better discrimination on the digital span and building block tests (mean accuracy of 83.4%; Table 2), suggesting that early cognitive impairment is primarily associated with declines in memory, attention and executive function (Petersen, 2004). In addition, statistical hypothesis testing analyses found that time features performed more prominently on cognitive assessments; in other words, mobile apps could capture favorable cognitive parameters more efficiently than traditional paper-based screening methods. Notably, two tests (i.e., the color interference and listening test) performed poorly in screening the MCI, providing insights for future cognitive systems improvements.

Third, regarding the application of wearable sensors in MCI validation, the combination of the three modalities achieved an accuracy of 86.2% (Table 4). In other words, the best classification results were obtained by combining selected features of all modalities compared to the separate modalities. Furthermore, HRV reflects the activity of the autonomic nervous system and can be used as a parameter to monitor the health status of the elderly. Assessment of HRV characteristics showed that SDNN, pNN50 and LF were the most important predictors (Table 2), implying that sympathetic autonomic regulation contributes well to overall cognitive function (Dalise et al., 2020). The autonomic nervous system has been reported to be activated during cognitive assessment (Nicolini et al., 2014; Luque-Casado et al., 2016). However, participants’ EDA values were significantly higher during the task than resting state (Figure 3), reflecting that their mental health may be dominated by stress or an increased awareness of the ongoing job. High arousal levels are a complex physiological response to stress and task awareness observed during daily activities, accompanied by increased EDA and reduced heart rate (Hernandez et al., 2014). Finally, our findings also suggest that EEG recorded by MUSE has the potential to screen for neurodegenerative diseases. For example, (1) the MCI group showed an increase in power spectral density at slower frequencies (i.e., delta and theta) compared to controls (Figure 3); (2) EEG features selected by RFECV method were concentrated in the alpha and beta bands (Table 2), indicating significant differences between the investigated groups in these two bands; (3) Mean power was most frequent in distinguishing MCI patients from healthy individuals (Table 2), in other words, lower mean power indicated a significant association with poorer cognitive performance on psychometric tests (Luckhaus et al., 2008).

Finally, some limitations and future directions can be worked on in this paper. First, our sample was relatively small, including only 120 participants in the validation process. Transfer learning provides an idea for solving the small sample problem (Rahaman et al., 2020), and a richer dataset will enhance the validation of the screening effect of the MCI classification framework. Secondly, we ignored the relationship between physiological signals and cognitive domains (e.g., language, memory, attention, visuospatial and executive functions, etc.) to highlight whether these data or their derived features can be considered indicators of general cognitive functioning. In the next step, we will investigate participants’ physiological data under different cognitive domains to assess whether they can be regarded as predictors of cognitive performance. Finally, our classification framework is based on traditional machine learning methods and in the future, as the sample increases, we propose to improve the classification performance further using deep learning models (Zhao et al., 2022; Chen et al., 2023).

## 5. Conclusion

This study used digital cognitive tests and multimodal wearable sensors (e.g., PPG, EDA and EEG) to screen for MCI. We acquired physiological signals from participants at rest and during the task, and extracted features from these data. We found that participants differed significantly in their physiological signals during these two phases (i.e., resting and task states). Moreover, data from multiple modalities provided better classification performance than data from either modality alone, implying that combining the two (i.e., mobile app and wearable device) can synergistically influence MCI screening. The RFECV method and weighted soft voting strategy provided 88.9% classification accuracy by performing 10-fold cross-validation using a selected subset of features. With the popularization of mobile computing platforms, our classification framework may provide new ideas and practical support for early MCI detection in home and portable screening.

## Data availability statement

The raw data supporting the conclusions of this article will be made available by the authors, without undue reservation.

## Ethics statement

The studies involving human participants were reviewed and approved by Biomedical Ethics Review Committee of Taiyuan University of Technology. The patients/participants provided their written informed consent to participate in this study.

## Author contributions

AL: thesis writing and experiments. JL and WW: data collection and processing. DZ and JZ: proofreading essay formatting and grammar. YQ: overall control of the thesis. All authors contributed to the article and approved the submitted version.

## Funding

This work was supported by the Central Guidance for Local Science and Technology Development Funds (YDZJSX2022C004) and National Natural Science Foundation Collaboration (U21A20469).

## Conflict of interest

The authors declare that the research was conducted in the absence of any commercial or financial relationships that could be construed as a potential conflict of interest.

## Publisher’s note

All claims expressed in this article are solely those of the authors and do not necessarily represent those of their affiliated organizations, or those of the publisher, the editors and the reviewers. Any product that may be evaluated in this article, or claim that may be made by its manufacturer, is not guaranteed or endorsed by the publisher.
