# Supplementary material for: Synergy through integration of digital cognitive tests and wearable devices for mild cognitive impairment screening
Source: Front Hum Neurosci. 2023 Apr 18;17:1183457. doi: 10.3389/fnhum.2023.1183457 (PMC10151757; doi:10.3389/fnhum.2023.1183457)
Supplement: Supplementary file 1 [file Presentation_1.pdf]

Details of physiological signal features

Table 1. Heart Rate (HR) and Heart Rate Variability (HRV) features.

Category	Name	Description	Unit
Statistical	min/max HR	Minimum and maximum of the HR	bpm
	mean HR	Mean of the HR_i	bpm
	median HR	Median of the HR_i	bpm
Time domain	SDNN	SD of all NN intervals	ms
	RMSSD	The square root of the mean of the sum of the squares of differences between adjacent NN intervals	ms
	NN_{50}	Number of pairs of adjacent NN intervals differing by more than 50 ms in the entire recording	ms
	pNN_{50}	NN_{50} count divided by the total number of all NN intervals	%
	NN_{20}	Number of pairs of adjacent NN intervals differing by more than 20 ms in the entire recording	/
	pNN_{20}	NN_{20} count divided by the total number of all NN intervals.	%
	CVNN	Coefficient of variation equal to the ratio of SDNN divided by mean NN interval	/
	CVSD	Coefficient of variation of successive differences equal to the RMSSD divided by mean NN interval	/
	mean	Mean of the IBIs	ms
	std	Standard deviation of the IBIs	ms
	min/max	Minimum and maximum of the IBIs	ms
	ptp	Range (peak to peak) of the IBIs	ms
	sum	Sum of the IBIs	ms
	energy	Energy of the IBIs	ms^2
	skewness	Skewness of the IBIs	/
	kurtosis	Kurtosis of the IBIs	/
	peaks	Number of the IBIs	/
	rms	Root mean square of the IBIs	ms
	line_integral	Integral under the IBIs	ms
	n_above_mean	Number of IBIs above the mean	/
	n_below_mean	Number of IBIs below the mean	/
	n_sign_changes	Number of changes in the IBIs slope	/
	iqr	Interquartile range between the 25th and 75th percentile of the IBIs	ms
	iqr 5_95	Interquartile range between the 5th and 95th percentile of the IBIs	ms
	pct 5	5th percentile of the IBIs	/
	pct 95	95th percentile of the IBIs	/
	entropy	Entropy of the IBIs	/
	perm entropy	Permutation entropy of the IBIs	/
	svd entropy	Singular value decomposition of the IBIs entropy	/
Frequency domain	total power	The variance of NN intervals over the temporal segment below 0.04 Hz	ms^2
	vlf	Power in very low frequency range below or equal 0.04 Hz	ms^2
	lf	Power in low frequency range 0.04 Hz and 0.15 Hz	ms^2
	hf	Power in high frequency range 0.15 Hz and 0.4 Hz	ms^2
	lf/hf_ratio	Ratio of LF to HF	/
	lfnu	LF power in normalized units	/
	hfnu	HF power in normalized units	/

High Frequency (HF), Low Frequency (LF), Inter-Beat Interval (IBI), and Normal-to-Normal (NN) interval.

Table 2. Electrodermal Activity (EDA) features.

Category	Name	Description	Unit
Time domain	mean	Mean of the SCR and SCL	μS
	std	Standard deviation of the SCR and SCL	μS
	min/max	Minimum and maximum of the SCR and SCL	μS
	ptp	Range (peak to peak) of SCR and SCL within a time interval	μS
	sum	Sum of the SCR and SCL values with a time interval	μS
	energy	Energy of the SCR and SCL	μS^2
	skewness	Skewness of the SCR and SCL	/
	kurtosis	Kurtosis of the SCR and SCL	/
	peaks	Number of SCR and SCL peaks with a time interval	/
	rms	Root mean square of the SCR and SCL	μS
	line_integral	Integral under the SCR and SCL curve	μS.s
	n_above_mean	Number of SCR and SCL data-points above the mean	/
	n_below_mean	Number of SCR and SCL data-points below the mean	/
	n_sign_changes	Number of changes in the SCR and SCL slope	/
	iqr	Interquartile range between the 25th and 75th percentile of the SCR and SCL	μS
	iqr_5_95	Interquartile range between the 5th and 95th percentile of the SCR and SCL	μS
	pct_5	5th percentile of the SCR and SCL	/
	pct_95	95th percentile of the SCR and SCL	/
	entropy	Entropy of the SCR and SCL	/
	perm_entropy	Permutation entropy of the SCR and SCL	/
	svd_entropy	Singular value decomposition of the SCR and SCL entropy	/
Frequency domain	sma	Signal magnitude area of the frequency domain SCR and SCL	μS
	energy	Energy of the frequency domain SCR and SCL	μS^2
	kurtosis	Kurtosis of the frequency domain SCR and SCL	/
	iqr	Interquartile range of the frequency domain SCR and SCL	μS/Hz
	spectral_power	5 spectral power magnitudes in the [0.05-0.55] Hz bands for the power density of the SCR and SCL	μS^2/Hz
	var_power	Variance of the SCR and SCL spectral power	μS^2
Time-frequency domain	mean	Mean of the SCR's and SCL's MFC signal	μS
	std	Mean of the SCR's and SCL's MFC signal	μS
	median	Median of the SCR's and SCL's MFC signal	μS
	iqr	Interquartile range of the SCR's and SCL's MFC signal	μS
	skewness	Skewness of the SCR's and SCL's MFC signal	/
	kurtosis	Kurtosis of the SCR's and SCL's MFC signal	/
SCR time-domain features	peaks	Number of SCR peaks	/
	rise_time	Mean of the SCR peaks rise time	s
	max_deriv	Mean value of the maximum derivative of the SCR peaks	μS/s
	amp	Mean amplitude of the SCR peaks	μS
	decay_time	Mean of the SCR peaks decay time	s
	scr_width	Mean width of the SCR peaks	s
	auc_mean	Mean area-under-curves of the SCR peaks	μS.s
	auc_sum	Sum of the area-under-curves of the SCR peak	μS.s

Mel-Frequency Cepstrum (MFC), skin conductance level (SCL), and skin conductance response (SCR).
